# The Impact of Small Incision Lenticule Extraction on the Biomechanical Properties of the Cornea: A Review

**DOI:** 10.3390/bioengineering12111199

**Published:** 2025-11-03

**Authors:** Yifan Du, Hanyu Jiang, Fei Mo, Yang Jiang

**Affiliations:** 1 Department of Ophthalmology, Peking Union Medical College Hospital, Peking Union Medical College, Chinese Academy of Medical Sciences, No. 1 Shuaifuyuan, Dongcheng District, Beijing 100730, China; duyifan260930@163.com (Y.D.);; 2 Key Laboratory of Ocular Fundus Diseases, Peking Union Medical College, Chinese Academy of Medical Sciences, Beijing 100006, China

**Keywords:** biomechanics, cornea, small incision lenticule extraction, refractive error, corneal thickness

## Abstract

Recently, due to advancements in keratorefractive surgery, small incision lenticule extraction (SMILE) has become increasingly recognized as a top surgical technique for treating refractive defects. The technology employs a femtosecond laser to precisely incise a stromal lenticule, which is subsequently extracted through a small incision, thereby preserving the front and most rigid regions of the cornea with minimal damage. Despite the widespread recognition of SMILE for its safety, biomechanical stability, effectiveness, and predictability, studies consistently document occurrences of postoperative keratectasia and a notable reduction in corneal biomechanical stability following the surgery. Hence, it is imperative to conduct further research on the several parameters linked to corneal biomechanical stability following SMILE. This narrative review comprehensively synthesizes the current literature on this topic and examines the literature on the evaluation of corneal biomechanics before and after SMILE. It provides a thorough review of the fundamental principles of corneal biomechanics, measurement techniques, the impact of various keratorefractive surgeries on corneal biomechanics, and the mechanisms through which SMILE affects corneal biomechanics.

## 1. Introduction

Small incision lenticule extraction (SMILE) is an innovative keratorefractive surgical procedure that employs femtosecond laser photolysis to generate a precisely shaped disc of corneal stromal tissue. This lenticule is subsequently eliminated by a diminutive incision measuring 2–4 mm. The complete procedure does not necessitate the creation of a corneal flap, and it allows for the preservation of the structure of the front part of the cornea [1,2]. The improvement in symptoms of corneal irritation and the stability of corneal biomechanics was superior. SMILE provides similar visual outcomes and fewer epithelial flap complications and dry eye incidences than laser-assisted in situ keratomileusis (LASIK) [3].

Corneal biomechanics refers to the study of the cornea’s mechanical behavior, including its strength, elasticity, and ability to maintain shape under pressure. They are clinically crucial as they determine the cornea’s ability to maintain its structural integrity, withstand intraocular pressure, and preserve refractive stability. Alterations in these properties can lead to serious complications such as keratectasia, highlighting the importance of thorough preoperative assessment and individualized surgical planning.

Nevertheless, cases of keratoconus and postoperative keratectasia have been documented following SMILE, yet the underlying causes remain unknown. Keratectasia is a complication that can occur after refractive surgery [4], but its occurrence is rare, with a reported prevalence of approximately 0.04–0.6%. Nevertheless, keratectasia poses a significant risk to eyesight and may necessitate corneal transplantation in extreme instances [5,6]. Nevertheless, changes in the mechanical characteristics of the cornea can be identified prior to the development of keratectasia, presenting as modifications in the shape of the cornea [7]. Multiple studies have demonstrated associations between alterations in corneal biomechanical stability and the refractive state before SMILE, the depth of tissue removal during the procedure, and the changes in corneal shape after the surgery [8]. Nevertheless, the specific pattern of corneal biomechanical alterations caused by SMILE is still unclear and can be affected by various other circumstances.

This review distinguishes itself from existing literature by providing a comprehensive, up-to-date synthesis of SMILE-specific biomechanical changes, systematically comparing multiple keratorefractive procedures, and incorporating recent advances in measurement technologies. We add to the field by critically evaluating conflicting evidence, offering clinical insights on key surgical parameters, and proposing future research directions to optimize patient outcomes.

Therefore, this study aimed to further explore the causes of changes in corneal biomechanics after SMILE by providing a review of the basic concepts, measurements, and comparisons of different keratorefractive surgeries. The mechanisms by which SMILE affects corneal biomechanics have been explored.

## 2. Methods

### 2.1. Review Type and Rationale

This article is a narrative review that aims to synthesize and critically appraise the existing literature on the impact of SMILE on corneal biomechanics. A narrative approach was chosen to provide a comprehensive, thematic overview of a broad and rapidly evolving field, encompassing diverse study designs, measurement technologies, and surgical parameters.

### 2.2. Literature Search Strategy

A comprehensive literature search was conducted in the electronic databases PubMed, Web of Science, and Google Scholar for articles published between January 2014 and June 2024. The search terms included “SMILE,” “small incision lenticule extraction,” “corneal biomechanics,” “Corvis ST,” “ORA,” “keratectasia,” “cap thickness,” and “residual stromal bed.” Boolean operators (AND, OR) were used to combine these terms.

### 2.3. Inclusion and Exclusion Criteria

The inclusion criteria were (1) original research articles reporting corneal biomechanical changes after SMILE; (2) studies comparing SMILE with other refractive procedures; (3) articles discussing corneal biomechanics measurement techniques; (4) studies published in English. Exclusion criteria included (1) editorials, conference abstracts, and case reports with fewer than 10 patients; (2) studies without full-text access; (3) articles not focusing on biomechanical outcomes.

### 2.4. Study Selection and Data Synthesis

Titles and abstracts were screened for relevance, and full-text articles were reviewed for final inclusion. Data were extracted and synthesized thematically, focusing on measurement techniques, comparative outcomes, and factors influencing biomechanical changes.

## 3. Results

### 3.1. Overview of Corneal Biomechanics

#### Basic Concepts of Corneal Biomechanics

Corneal biomechanics is a field that combines different disciplines to quantitatively analyze the cornea using mechanical principles [9]. The biomechanical characteristics of the cornea are intricately connected to its structure, which plays a crucial role in determining its capacity to refract light. Pathologies, traumas, or surgical procedures involving the cornea can have a negative impact on its biomechanical properties and may also cause a certain degree of visual impairment [10]. The cornea is positioned anteriorly in the eye and serves as a refractive medium. Its primary functions include maintaining stable intraocular pressure and safeguarding intraocular tissues against harm [11]. The cornea possesses viscoelastic properties, and the reticular fibers inside the corneal stroma play a crucial role in preserving the cornea’s shape and rigidity [12]. Several corneal structures have been discovered to impact the biomechanical qualities of the cornea to a certain degree. The stroma, which constitutes almost 90% of the corneal thickness, is very valuable in studying corneal biomechanics. The quantity and arrangement of collagen fibers and extracellular matrix play a crucial role in preserving the biomechanical properties of the cornea. The consensus is that the front stroma has a greater impact on biomechanics compared to the posterior stroma, whereas the peripheral stroma has a greater influence on corneal biomechanics than the central stroma [9,13]. The endothelium, a crucial component of the corneal atrial water barrier, might indirectly impact corneal rigidity and, consequently, corneal biomechanics by regulating the water content of the stroma [9].

### 3.2. Measurements of Corneal Biomechanics

To date, there are two main types of corneal biomechanical measurements: ex vivo and in vivo measurements. Ex vivo measurements mainly include corneal axial stretch tests, corneal expansion tests, and ex vivo whole eye measurements [14]; In vivo measurements mainly include ocular response analysis (ORA), corneal visualization with Scheimpflug Technology (Corvis ST), Brillouin Optical Microscopy (BOM), optical coherence elastography (OCE), Supersonic Shear-Wave Imaging (SSWI), and phase-decorrelation optical coherence tomography (PhD-OCT) [15], of which ORA and Corvis ST have been more widely used in clinical practice (Table 1). They can detect the overall corneal properties based on the jet method but lack the spatial resolution to detect local differences in corneal biomechanical properties, and are susceptible to corneal edema, corneal thickness, and intraocular pressure [16,17]. In contrast, newer measurement tools such as BOM, OCE, SSWI, and PhD-OCT have shown the potential to measure corneal biomechanics, while a higher spatial resolution makes it easier to identify areas of abnormality [18,19,20]. Although there are still many obstacles to applying these new methods in the clinic, such as eye movement artifacts, degree of corneal hydration, instrumental limitations, and imaging speed, it is undeniable that their numerous advantages will provide new opportunities for clinical corneal biomechanical measurements. In any case, the differences in these instruments, the errors in each measurement, and the non-directive approach all influence biomechanical measurements, which will lead to differences in assessment before and after SMILE.

### 3.3. Effects of Keratorefractive Surgery on Corneal Biomechanics

**Figure 1:**
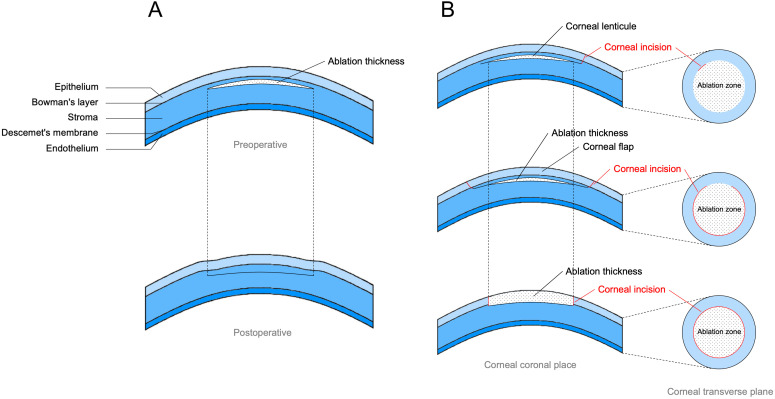
The process of keratorefractive surgery and its principles affecting corneal biomechanics. (**A**) The procedure of keratorefractive surgery in which a certain thickness is ablated from the corneal stroma, resulting in a thinning of the cornea and a consequent reduction in corneal refractive power. (**B**) The effect of different surgical procedures on corneal biomechanics, small incision lenticule extraction (SMILE) at the top, laser-assisted in situ keratomileusis (LASIK) in the middle, and photorefractive keratectomy (PRK) at the bottom, which have different incision sizes and therefore different disruptions to the Bowman’s Layer, which may lead to different biomechanical effects. This figure is original and was created for this review.

Modern keratorefractive surgery works by removing part of the corneal stroma to flatten the cornea, which, in turn, reduces the refractive power of the cornea to correct myopia. This inevitably leads to thinning of the cornea, reduction in the amount of stromal collagen, and disruption of collagen interconnections. Consequently, the biomechanical stability of the cornea is reduced. In the past, corneal thickness remaining after surgery was used as a criterion for assessing the likelihood of keratectasia after surgery, although this remains uncertain as keratectasia increases after keratorefractive surgery. Although corneal thickness is a more accurate indicator of tissue resistance to tension, the biomechanics of the cornea are not determined by corneal thickness alone. Previous studies have pointed out that keratorefractive surgery also damages the Bowman’s layer and anterior stromal layers of the cornea, which are considered key components of corneal tensile strength and are the main factors contributing to changes in corneal biomechanics [10]. Biomechanical changes in the cornea may be related to a number of factors, such as corneal morphology before surgery, different surgical methods chosen, and changes in corneal thickness after surgery [13]. Therefore, a thorough examination and evaluation of corneal biomechanics are necessary before performing keratorefractive surgery. For different surgical procedures, the depth of ablation and degree of disruption of Bowman’s layer can also have different effects on the biomechanics of the cornea. Figure 1 illustrates the process of keratorefractive surgery and its principles affecting the corneal biomechanics. 

### 3.4. SMILE and Corneal Biomechanics

#### The Effect of SMILE on Corneal Biomechanics

SMILE is a popular type of keratorefractive surgery that promotes corneal stability by eliminating the need for a corneal flap. Currently, it is regarded as a refractive surgery that has the least impact on the biomechanics. Nevertheless, there have been recorded instances of postoperative keratectasia [4]. Thus, SMILE should prioritize the examination of factors that influence biomechanical alterations in the cornea. The impact of corneal cap thickness on postoperative biomechanics remains a subject of debate, with studies reporting seemingly conflicting results. For instance, Wu et al. [21,22] reported that thinner caps (110–120 μm) were associated with smaller alterations in parameters like A2T, DA, and IR, or higher overall biomechanical strength, compared to thicker caps (140 μm). This suggests that preserving a greater amount of the anterior stroma, which is biomechanically stronger, might be beneficial. However, this finding is not universal. Other studies [23,24] found no statistically significant differences in biomechanical parameters between groups with cap thicknesses of 110 μm versus 160 μm, or 110 μm versus 150 μm, respectively. These discrepancies may be attributed to variations in study populations, including preoperative corneal thickness, the degree of myopia corrected, and sample size. Supporting the theory of anterior stromal importance, other researchers demonstrated that a 160 μm cap had a significantly smaller impact on biomechanics than a 100 μm cap [25]. Collectively, these studies indicate that the absolute cap thickness may not be the sole determinant; rather, the relative proportion of the anterior stroma preserved (e.g., cap thickness relative to total corneal thickness) is likely a more critical factor influencing postoperative biomechanical stability and warrants further investigation. Table 2 displays the most recent 5 years of research on corneal biomechanical alterations after SMILE [21,24,26,27,28,29,30,31,32,33,34,35,36,37]. 

### 3.5. Comparison of Corneal Biomechanical Effects of Other Keratorefractive Surgery Versus SMILE

#### 3.5.1. PRK/LASEK vs. SMILE

Studies have found conflicting evidence regarding the biomechanical effects on the cornea after surface surgeries like photorefractive keratectomy (PRK), transepithelial PRK (trans-PRK), or laser-assisted subepithelial keratomileusis (LASEK) compared to SMILE. Liu et al. [38] reported this controversy in their research. Guo et al. [39] found that following PRK or LASEK procedures, CH and CRF in ORA exhibited considerably higher values compared to SMILE. Nevertheless, in the majority of the analyzed trials, the quantity of tissue extracted using SMILE surpassed that of PRK/LASEK. This discrepancy might be attributed to the fact that SMILE was employed to treat more severe cases of myopia compared to surface surgery techniques. Hence, direct comparison of absolute biomechanical changes between SMILE and surface ablation can be confounded by differences in the amount of tissue removed. To enable a more meaningful comparison, future studies should normalize biomechanical changes to the degree of correction (per dioptre) or the volume of tissue extracted. An example of this approach is the study by Yu et al. [30], which compared the change in CH and CRF per unit of corneal tissue removed and found SMILE to be biomechanically superior to LASEK in the early postoperative period. However, these disparities became essentially insignificant over an extended duration. Shen et al. [40] conducted a comprehensive analysis of the corneal biomechanical parameters of SMILE and LASEK utilizing Corvis ST. The findings revealed that there were no notable changes between the two procedures.

#### 3.5.2. FS-LASIK or FLEx Versus SMILE

Multiple scholarly articles have provided evidence that the biomechanical performance following SMILE is notably superior to that of LASIK or femtosecond-LASIK (FS-LASIK) [31,32,41,42]. Guo et al. [39] conducted a meta-analysis that showed that the values of CH and CRF obtained through SMILE in ORA were significantly higher than those obtained with FS-LASIK. This finding aligns with the outcomes of a meta-analysis conducted by Yan et al. [43] based on five investigations. Wang et al. [44] assessed the alterations in posterior corneal elevation and corneal biomechanical characteristics in individuals with high myopia who underwent SMILE and FS-LASIK. Their findings demonstrated that SMILE outperformed FS-LASIK in preserving the stability of the posterior corneal surface at the 12-month mark following the surgical procedure. The authors proposed that SMILE may offer significant benefits in the field of biomechanics, namely in the treatment of severe nearsightedness. The meta-analysis conducted by Raevdal et al. [45] revealed a noteworthy reduction in corneal biomechanics, as assessed by ORA, across several types of procedures such as SMILE, FLEx, and FS-LASIK. The decline in corneal biomechanics was notably more pronounced in the FS-LASIK group compared to the SMILE group. Nevertheless, multiple randomized controlled trials have indicated that there is no substantial disparity in CH or CRF between SMILE and keratorefractive surgery with a corneal flap (FLEx or FS-LASIK) [23,46,47]. The results align with the findings of Vestergaard et al. and Agca et al. [23,48], who demonstrated that there were no notable biomechanical distinctions between the SMILE and FS-LASIK or FLEx techniques. Based on a thorough investigation, they have formulated a hypothesis that several factors may be responsible for the variation in results. These factors include the impact of the measurement apparatus, intraocular pressure (IOP), central corneal thickness (CCT), degree of corrected refractive error, and age [49]. Kanellopoulos et al. [50] found that SMILE resulted in a more significant reduction in tensile strength compared to LASIK in eyes with low myopia. However, in highly myopic eyes, both SMILE and LASIK were associated with a greater decrease in tensile strength compared to FS-LASIK, according to Wei et al. [51]. The study conducted by Kanellopoulos et al. [50] found that both SMILE and LASIK procedures resulted in a comparable reduction in corneal strength. Wei et al. [51] conducted a study to assess the alteration in biomechanical characteristics per unit of corneal volume altered. They discovered that the extent of change following SMILE was less than that observed after FS-LASIK, providing evidence for SMILE’s superior biomechanical efficiency when the amount of tissue modification is accounted for. Hence, in order to conduct future studies involving paired-eye analysis and the collaboration of research teams, it is imperative to verify the biomechanical discoveries following SMILE as compared to FS-LASIK.

### 3.6. Exploration of Mechanisms Affecting Corneal Biomechanical Changes After SMILE

#### Changes in Corneal Thickness

Corneal thickness plays a crucial role in shaping the biomechanics of the cornea. Therefore, analyzing changes in corneal thickness and residual corneal thickness following SMILE is essential for assessing the stability of corneal biomechanics post-operation. This review examined three indicators: corneal residual bed thickness (RBT), corneal cap thickness, and lenticule thickness.

RBT refers to the thickness from the postablation surface to the posterior surface of the cornea. A decrease in RBT may make the cornea thinner, potentially leading to a decrease in the biomechanical stability of the cornea. Jia et al. [52] studied different RBT thicknesses after SMILE in rabbits and found that when RBT was low, there was a significant decrease in the first applanation velocity (A1V), second applanation velocity (A2V), DA, and IR within the 1st month after the procedure. Although corneal stiffness showed a negative correlation with RBT in the 1st month after surgery, most biomechanical parameters returned to their pre-surgical state in the 3rd month after surgery. In contrast, Liu et al. [53] found that RBT after SMILE negatively correlated with changes in certain corneal biomechanical parameters. They further noted that when the RBT was less than 280 μm, there were significant changes in key parameters, such as DAR and SP-A1, which served as an early warning for clinical practice. According to Wu et al. [22], there was a significant correlation between RBT and the ΔCRF index, which implies that there is a correlation between RBT and the decrease in biomechanical parameters after SMILE. However, there is no clear conclusion regarding the association between corneal biomechanical changes and corneal cap thickness following SMILE. In recent years, a study by Damgaard et al. [54] pointed out that a corneal cap of 110μm may trigger more severe biomechanical changes compared with 160 μm, but the results of these two groups did not show a significant difference. A study by Liu et al. [55] demonstrated that three months after SMILE, biomechanical indices between the 110 μm group and the 150 μm group were not significantly different. However, Massry et al. [56] found that the use of microlenses at a depth of 160 μm had a much smaller effect on corneal biomechanics than at 100 μm during SMILE. This confirms that the anterior portion of the corneal stroma contributes more to corneal biomechanics than the posterior portion, which is similar to the selection of corneal cap thickness. Meanwhile, Jun et al. [24] pointed out that corneal biomechanical attenuation was more significant in the 140 μm group than in the 120 μm group. The thickness of the lenticule, on the other hand, depends mainly on the degree of refractive error to be corrected, i.e., the greater the degree of refractive error, the thicker the lenticule is. The scientific team of Hosny et al. [57] observed that there was a significant reduction in both CH and CRF after SMILE, and the extent of this reduction was positively correlated with the thickness of the lenticule used in the procedure; there was a significant correlation. This is similar to the thickness of RBT, in which a thinner RBT may result in poorer biomechanical stability.

**Figure 2:**
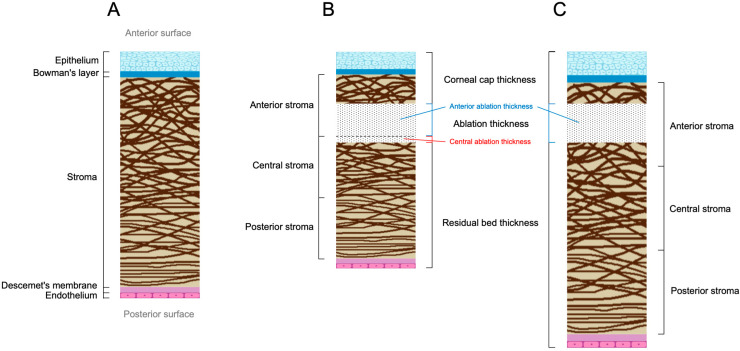
Thickness structure of cornea and the effect of SMILE at different corneal thicknesses. (**A**) The name and thickness structure of each corneal layer demonstrates that the collagen in the corneal stroma is arranged with a certain regularity, showing a decrease in the diameter of the collagen from the front to the back, a decrease in the degree of inclination, and a weakening of the interspersions and connections between the collagen. (**B**,**C**) show the ablation of the stroma during SMILE in thin corneal thicknesses (**B**) and thick corneal thicknesses (**C**). It can be seen that even when choosing the same corneal cap thickness and ablation thickness, the ablated corneal tissue varies from one thickness to the next, resulting in different biomechanical changes after SMILE. This figure is original and was created for this review.

However, it should be noted that the effect of corneal thickness changes on corneal biomechanics is not only determined by the above factors, but also by the preoperative corneal thickness also plays a key role, which may lead to significant inconsistencies when the above factors are the same, which may explain some of the inconsistent findings. Therefore, avoiding the effect of corneal thickness by means of ratios (e.g., RBT/corneal thickness) to avoid the effect of corneal thickness will be more relevant in future studies. Meanwhile, since the collagen arrangement and thickness of the corneal stroma changes from anterior to posterior, studying the thickness of the anterior and central corneal stromal ablation or using a ratio (e.g., anterior stromal ablation thickness/corneal thickness, central stromal ablation thickness/corneal thickness, or central stromal ablation thickness/anterior stromal ablation thickness) may provide a more realistic reflection of the association between them. Figure 2 shows the thickness structure of the corneal layers and the effect of SMILE at different corneal thicknesses.

### 3.7. Other Factors

Certain academic studies have pointed out that corneal volume (CV) is not only a metric used to comprehensively assess the overall actual changes in corneal tissue but is also a unique metric used to describe changes in corneal morphology [58]. According to Wei et al. [51] the SMILE group showed a decrease in the CV in its periphery (area between 5 and 10 mm in diameter) during the first week after surgery. Three months after surgery, changes in CV in different regions correlated with changes in CRF and CH, a finding that revealed a strong link between CV and corneal biomechanics. Liu et al. [59] found a correlation between the biomechanical parameters of the cornea and changes in CV in different regions. In particular, 3 months after surgery, the biomechanical parameters changed significantly compared to those before surgery. Additionally, CV has the potential to be an important predictor of postoperative corneal expansion, thus providing an important reference for clinical practice.

The opaque bubble layer (OBL) is a phenomenon that occurs when using a femtosecond laser to create a corneal lenticule during SMILE, where gas bubbles accumulate within the corneal stroma. Instead of fully disappearing, the bubbles created by the laser gather together between the collagen fibers of the corneal stroma, forming clusters of bubbles (bubble aggregates) [3]. A study conducted by Ma et al. [60] discovered a notable disparity in corneal biomechanics-related factors between the OBL and no-OBL groups. This suggests that the development of OBL is strongly associated with the biomechanical characteristics of the cornea. Consequently, it is crucial to implement suitable preventive measures during the surgical planning phase. Wu et al. [22] found that thickening the corneal cap can potentially decrease the occurrence of OBL in individuals who undergo SMILE for low-to-moderate myopia.

## 4. Discussion

This comprehensive review synthesizes current evidence on the impact of SMILE on corneal biomechanical properties, highlighting both the advantages of this flapless procedure and the factors contributing to postoperative biomechanical changes. Our analysis demonstrates that while SMILE better preserves corneal biomechanical strength compared to flap-based procedures like LASIK, it still induces measurable alterations in corneal hysteresis, resistance factor, and deformation parameters.

The conflicting evidence regarding SMILE compared to surface ablation procedures (PRK/LASEK) underscores the complexity of corneal biomechanical assessment. Discrepancies in study outcomes may be attributed to variations in surgical parameters, measurement techniques, and patient characteristics. Similarly, the comparison between SMILE and FS-LASIK reveals ongoing debate, with some studies favoring SMILE’s biomechanical profile while others show comparable outcomes.

Our exploration of mechanistic factors identifies corneal thickness parameters (RBT, cap thickness, lenticule thickness) as critical determinants of postoperative biomechanical stability. The anterior corneal stroma’s greater contribution to biomechanical strength explains why cap thickness selection significantly influences outcomes. Additionally, emerging factors such as corneal volume and opaque bubble layer formation provide new insights into the multifactorial nature of SMILE’s biomechanical effects.

A significant limitation in the current body of literature, as highlighted by this review, is the prevalent lack of normalization of biomechanical changes to the magnitude of surgical intervention. Comparisons of absolute changes in CH, CRF, or Corvis ST parameters between different studies or surgical techniques are of limited value without accounting for the corrected refractive error or the volume of stromal tissue removed. The findings of Wei et al. [51] and Yu et al. [30], who employed normalization techniques, provide a more nuanced and comparable insight. Future research must adopt standardized reporting metrics, such as change in biomechanical parameter per dioptre of correction or per micrometre of tissue ablation, to draw more valid and clinically relevant conclusions.

## 5. Limitations

This review has several limitations that should be acknowledged. First, as a narrative review, it does not employ systematic methodology or meta-analysis, which may introduce selection bias. Second, the included studies exhibit considerable heterogeneity in design, measurement tools, surgical protocols, and follow-up duration, limiting direct comparability. Second, the included studies exhibit considerable heterogeneity in design, measurement tools, surgical protocols, and follow-up duration, limiting direct comparability. Third, most available studies report short- to mid-term outcomes, while long-term biomechanical data after SMILE remain scarce. Fourth, the influence of confounding factors such as intraocular pressure fluctuations, corneal hydration status, and measurement artifacts cannot be fully discounted. Finally, the rapid evolution of measurement technologies and surgical techniques means that newer developments may not be fully captured in this review.

## 6. Conclusions

In summary, SMILE induces quantifiable alterations in corneal biomechanical properties, the extent of which is modulated by a complex interplay of surgical parameters, including residual stromal bed thickness, cap thickness, and lenticule thickness. Theoretical advantages and a substantial body of evidence suggest that SMILE may better preserve corneal biomechanical strength compared to flap-based procedures like LASIK, likely due to the absence of a flap and reduced disruption to the anterior stromal lamellae. However, the evidence is not entirely consistent, and comparisons with surface ablation techniques remain inconclusive, particularly when normalization for the degree of correction is not applied. Although rare, postoperative keratectasia has been reported after SMILE, underscoring the imperative of rigorous preoperative screening. Therefore, while SMILE represents a valuable and often biomechanically favorable option in the refractive surgery arsenal, it should not be considered universally superior without consideration of individual patient factors. Future long-term studies employing standardized, normalized outcomes are essential to precisely define SMILE’s biomechanical impact and optimize personalized surgical planning.

## Figures and Tables

**Table 1:** Comparison of corneal biomechanics measured by different instruments.

	Measurement Principles	Measurement Process	Main Observed Indicators	Advantages	Disadvantages
ORA	The bi-directional movement of the cornea as well as the deformation of the cornea is monitored by the reflection of the infrared beam	A rapid pulse of air is applied to a 3–6 mm area in the centre of the cornea to measure the process of inward depression of the cornea due to the pulse of air, flattening of the cornea to form a slight depression, and rebound of the cornea back to its normal state	CH, CRF, and 37 new parameters describing ORA curve waveforms	Non-contact, rapid and more accurate detection of corneal biomechanics	Does not directly provide standardised biomechanical indications of the cornea, does not reflect the specific location and severity of the cornea, and may not reflect the biomechanical tensile strength of the cornea in its physiological state when measured by indentation
Corvis ST	Corneal deformation was monitored using an ultra-high speed Scheimpflug camera (4300 frames per second)	Similarly to ORA, a pulse of air with a maximum pressure of 25 kPa was applied, and the process of corneal inward depression due to the action of the pulsed air flow, flattening and then forming a slight depression, and corneal rebound back to its normal state was measured	DA, HCPD, SP-A1, IR, DAR, CBI, and other parameters	Non-contact, rapid and more accurate detection of corneal biomechanics, higher visualisation compared to ORA	Same as ORA
OCE	An elastography method developed based on optical coherence tomography	(1) inducing soft tissue deformation or vibration; (2) detecting the propagation of deformation, displacement, vibration, or oscillation; (3) assessing soft tissue elasticity	Modulus of elasticity, which means that the measured data on the elastic response of the tissue is mathematically modelled and finally presented in the form of Young’s modulus	Non-invasive imaging, real-time image processing performance and high resolution	Lack of harmonised clinical standards and non-existence of commercially available devices
BOM	Based on the Brillouin scattering principle	A low-power focused laser beam and a high-resolution confocal spectrometer were used to measure the Brillouin frequency at the focal point	Differences are shown by comparing the Brillouin frequency shift of the cornea, but there is no uniformity in the current range of reference values	Good spatial resolution, no contact during imaging, no external loads required	Long collection time, highly influenced by the degree of corneal hydration, and the lack of commercially available equipment
SSWI	Elastography based on ultrasound imaging	Quantification of transverse wave velocities generated by focused ultrasound in tissues using high frame rate (up to 20,000 frames/s) ultrasound imaging and linking them to elastic moduli through mathematical modelling	Modulus of Elasticity. Similar to OCE, through mathematical modelling, and ultimately in the form of Young’s modulus	Real-time mapping of tissue elasticity and good spatial resolution	Requires liquid coupling medium to contact the cornea, long image acquisition times, and complex data processing
PhD-OCT	Corneal biomechanics measurements based on dynamic light scattering theory	Image acquisition is achieved using two OCT devices with different centre wavelengths, and post-acquisition processing is performed using the Fourier Transform	The parameter Γ. For the cornea, the parameter Γ is inversely proportional to the degree of collagen restriction. That is, the higher the parameter Γ, the lower the biomechanics	Good soft-tissue spatial resolution without any perturbation of the cornea, relatively simple measurements, less affected by IOP, short acquisition time, can be achieved using existing clinical OCT systems	High signal-to-noise ratio, susceptible to corneal hydration and eye movement artefacts

BOM, Brillouin optical microscopy; CBI, Corvis biomechanical index; CH, cornea hysteresis; Corvis ST, corneal visualization with Scheimpflug technology; CRF, cornea resistance factor; DA, deformation amplitude; DAR, deformation amplitude ratio; HCPD, highest concavity peak distance; IR, integrated inverse radius; OCE, optical coherence elastography; ORA, ocular response analyzer; PhD-OCT, phase-decorrelation optical coherence tomography; SP-A1, stiffness parameter at first applanation; SSWI, supersonic shear-wave imaging.

**Table 2:** Studies investigating corneal biomechanical changes following SMILE in the last 5 years.

Study	Eyes (n)	Country	Age (y)	Sphere (D)	Cylinder (D)	CCT (μm)	Follow-Up	Instruments	Assessment Parameters
Fu et al. [26]	13	China	32.8 ± 9.0	4.17 ± 1.55	−0.90 ± 0.75	546.7 ± 25.3	1 w, 1 m, 3 m	ORA, Corvis ST	bIOP, CH, CRF, A1T, DA, SP-A1
Cao et al. [27]	80	China	26.9 ± 5.6	−4.67 ± 1.31 *		539.8 ± 27.0	3 m	Corvis ST	bIOP, IR, HCR, DAR1, DAR2
Qu et al. [28]	69	China	27.3 ± 5.8	−5.17 ± 1.80	−0.74 ± 0.57	552.5 ± 24.2	3 m, 6 m	Corvis ST	DAR2, IR, SP-A1, HCT, SSIv2
Abd et al. [29]	30	Egypt	26.4 ± 5.4	−4.95 ± 1.32 *		556.8 ± 28.4	3 m	Corvis ST	bIOP, A1V, A2V, DAR, IR, ARTH, SP-A1, CBI
Yu et al. [30]	32	China	23.4 ± 4.6	−4.1 ± 0.8 *		551.1 ± 23.1	3 m, 3 y	ORA	IOPcc, CH, CRF
He et al. [31]	50	China	25.9 ± 4.9	−7.96 ± 1.11	−1.03 ± 0.69	552.8 ± 38.8	1 m, 3 m, 6 m, 15 m	Corvis ST	A1T, A1L, A1V, A2T, A2L, A2V, HCT, HCR, HCPD, DAR2, IR, ARTH, SP-A1, CBI, SSI
Xin et al. [32] (SMILE)	72 *	China	26.9 ± 5.7	−5.93 ± 1.05	−0.84 ± 0.49	547.0 ± 22.7	1 m, 3 m, 6 m	Corvis ST	SP-A1, IR, DA, DAR2
Xin et al. [32] (FS-LASIK)	72 *	China	25.7 ± 6.3	−3.38 ± 0.72	−0.81 ± 0.77	546.0 ± 20.3	1 m, 3 m, 6 m	Corvis ST	SP-A1, IR, DA, DAR2
Jun et al. (120 μm corneal cap) [24]	91	Korea	27.8 ± 6.0	−3.18 ± 1.28	−0.93 ± 0.69	565.2 ± 24.4	6 m	Corvis ST	DAR, SP-A1, IR, ARTH, SSI, bIOP
Jun et al. (140 μm corneal cap) [24]	59	Korea	27.3 ± 7.0	−3.26 ± 1.49	−0.97 ± 0.94	563.9 ± 22.0	6 m	Corvis ST	DAR, SP-A1, IR, ARTH, SSI, bIOP
Ghanavati et al. [33]	37	Iran	32.3 ± 6.7	−3.70 ± 1.92	−0.93 ± 0.93	523.8 ± 37.8	3 m	Corvis ST	bIOP, A1T, A1L, A1V, A2T, A2L, A2V, SP-A1, SP-HC, DA, DAmax, DAR2, HCT, HCPD, HCR, IRmax, ARTH, SSI, WEMmax
Lv et al. (110 μm corneal cap) [34]	48	China	24.0 ± 4.4	−5.02 ± 0.99	−0.55 ± 0.51	545.3 ± 15.0	1 w, 1 m, 3 m, 6 m	Corvis ST	IR, DAR1, DAR2, ARTH, SP-A1, SSI, bIOP
Lv et al. (120 μm corneal cap) [34]	49	China	25.5 ± 4.4	−4.87 ± 1.01	−0.79 ± 0.69	546.6 ± 15.0	1 w, 1 m, 3 m, 6 m	Corvis ST	IR, DAR1, DAR2, ARTH, SP-A1, SSI, bIOP
Lv et al. (130 μm corneal cap) [34]	49	China	24.0 ± 4.1	−4.60 ± 0.82	−0.75 ± 0.81	551.3 ± 9.9	1 w, 1 m, 3 m, 6 m	Corvis ST	IR, DAR1, DAR2, ARTH, SP-A1, SSI, bIOP
Liu et al. [35]	45	China	25.2 ± 6.5	−4.99 ± 1.06		544.7 ± 20.8	1 m, 6 m	Corvis ST	IR, DAR2, ARTH, SP-A1
Wu et al. (110 μm corneal cap) [21]	50	China	25.0 ± 5.1 *	−4.54 ± 0.95		551.2 ± 26.0	6 m	Corvis ST	bIOP, A1T, A1L, A1V, A2T, A2L, A2V, SP-A1, HCPD, HCR, DA, IR, DAR
Wu et al. (140 μm corneal cap) [21]	50	China	25.0 ± 5.1 *	−4.50 ± 1.03		550.9 ± 25.1	6 m	Corvis ST	bIOP, A1T, A1L, A1V, A2T, A2L, A2V, SP-A1, HCPD, HCR, DA, IR, DAR
Hashemi et al. [36]	120	Iran	28.0 ± 5.3	−4.66 ± 0.85 *		567.0 ± 25.3	3 m, 1 y	Corvis ST	SSI, SP-A1, IR, DAR1, DAR2
Sedaghat et al. [37]	62	Iran	26.4 ± 5.2	−3.73 ± 1.18 *		552.1 ± 25.0		ORA, Corvis ST	A1L, A1V, A2L, A2V, HCPD, HCR, DA, SP-A1, ARTH, IR, DAR, CH, CRF

A1T/L/V, first applanation time/length/velocity; A2T/L/V, second applanation time/length/velocity; ARTH, ambrosio relational thickness to the horizontal profile; bIOP, biomechanically corrected intraocular pressure; CBI, Corvis biomechanical index; CH, cornea hysteresis; CRF, cornea resistance factor; DA, deformation amplitude; DAR1/2, deformation amplitude ratio at 1 mm/2 mm; HCPD, highest concavity peak distance; HCR, highest concavity radius; HCT, highest concavity time; IOPcc, corneal compensated intraocular pressure; IR, integrated inverse radius; SP-A1, stiffness parameter at first applanation; SP-HC, stiffness parameter at highest concavity; SSI, corneal stress–strain index; SSIv2, updated stress–strain index; WEM, linear anterior–posterior movement of the whole eye following maximum displacement of the cornea. (*) as the spherical equivalent (SE). All refractive data are presented as spherical equivalent (Mean ± SD). All values are displayed as mean ± standard deviation.

## Data Availability

Data used in this review article are publicly available.

## References

[1] Huang G., Melki S. (2021). Small Incision Lenticule Extraction (SMILE): Myths and Realities. Semin. Ophthalmol..

[2] Blum M., Kunert K.S., Sekundo W. (2017). Historical overview of the clinical development of the small incision lenticule extraction surgery (SMILE). Klin. Monbl. Augenheilkd..

[3] Krueger R.R., Meister C.S. (2018). A review of small incision lenticule extraction complications. Curr. Opin. Ophthalmol..

[4] Santhiago M.R., Giacomin N.T., Smadja D., Bechara S.J. (2016). Ectasia risk factors in refractive surgery. Clin. Ophthalmol..

[5] Wolle M.A., Randleman J.B., Woodward M.A. (2016). Complications of Refractive Surgery: Ectasia After Refractive Surgery. Int. Ophthalmol. Clin..

[6] Sutton G., Lawless M., Hodge C. (2014). Laser in situ keratomileusis in 2012: A review. Clin. Exp. Optom..

[7] Bao F., Geraghty B., Wang Q., Elsheikh A. (2016). Consideration of corneal biomechanics in the diagnosis and management of keratoconus: Is it important?. Eye Vis..

[8] Bao F., Lopes B.T., Zheng X., Zheng X., Ji Y., Wang J., Elsheikh A. (2023). Corneal Biomechanics Losses Caused by Refractive Surgery. Curr. Eye Res..

[9] Chong J., Dupps W.J. (2021). Corneal biomechanics: Measurement and structural correlations. Exp. Eye Res..

[10] Wilson A., Marshall J. (2020). A review of corneal biomechanics: Mechanisms for measurement and the implications for refractive surgery. Indian J. Ophthalmol..

[11] Kling S., Hafezi F. (2017). Corneal biomechanics—A review. Ophthalmic. Physiol. Opt..

[12] Winkler M., Shoa G., Xie Y., Petsche S.J., Pinsky P.M., Juhasz T., Brown D.J., Jester J.V. (2013). Three-dimensional distribution of transverse collagen fibers in the anterior human corneal stroma. Investig. Ophthalmol. Vis. Sci..

[13] Ma J., Wang Y., Wei P., Jhanji V. (2018). Biomechanics and structure of the cornea: Implications and association with corneal disorders. Surv. Ophthalmol..

[14] Zhao Y., Hu G., Yan Y., Wang Z., Liu X., Shi H. (2022). Biomechanical analysis of ocular diseases and its in vitro study methods. Biomed. Eng. Online.

[15] Li F., Wang K., Liu Z. (2023). In Vivo Biomechanical Measurements of the Cornea. Bioengineering.

[16] Qin X., Yu M., Zhang H., Chen X., Li L. (2019). The Mechanical Interpretation of Ocular Response Analyzer Parameters. Biomed. Res. Int..

[17] Miao Y.Y., Ma X.M., Qu Z.X., Eliasy A., Wu B.W., Xu H., Wang P., Zheng X.B., Wang J.J., Ye Y.F. (2024). Performance of Corvis ST Parameters Including Updated Stress-Strain Index in Differentiating Between Normal, Forme-Fruste, Subclinical, and Clinical Keratoconic Eyes. Am. J. Ophthalmol..

[18] Prevedel R., Diz-Muñoz A., Ruocco G., Antonacci G. (2019). Brillouin microscopy: An emerging tool for mechanobiology. Nat. Methods.

[19] Lan G., Twa M.D., Song C., Feng J., Huang Y., Xu J., Qin J., An L., Wei X. (2023). In vivo corneal elastography: A topical review of challenges and opportunities. Comput. Struct. Biotechnol. J..

[20] Tanter M., Touboul D., Gennisson J.L., Bercoff J., Fink M. (2009). High-resolution quantitative imaging of cornea elasticity using supersonic shear imaging. IEEE Trans. Med. Imaging.

[21] Wu D., Liu C., Li B., Wang D., Fang X. (2020). Influence of Cap Thickness on Corneal Curvature and Corneal Biomechanics After SMILE: A Prospective, Contralateral Eye Study. J. Refract. Surg..

[22] Wu Z., Wang Y., Zhang J., Chan T.C.Y., Ng A.L.K., Cheng G.P.M., Jhanji V. (2017). Comparison of corneal biomechanics after microincision lenticule extraction and small incision lenticule extraction. Br. J. Ophthalmol..

[23] Vestergaard A.H., Grauslund J., Ivarsen A.R., Hjortdal J.Ø. (2014). Central corneal sublayer pachymetry and biomechanical properties after refractive femtosecond lenticule extraction. J. Refract. Surg..

[24] Jun I., Kang D.S.Y., Roberts C.J., Lee H., Jean S.K., Kim E.K., Seo K.Y., Kim T.I. (2021). Comparison of Clinical and Biomechanical Outcomes of Small Incision Lenticule Extraction With 120- and 140-µm Cap Thickness. Transl. Vis. Sci. Technol..

[25] Pniakowska Z., Jurowski P., Wierzbowska J. (2022). Clinical Evaluation of Corneal Biomechanics following Laser Refractive Surgery in Myopic Eyes: A Review of the Literature. J. Clin. Med..

[26] Fu D., Li M., Knorz M.C., Wei S., Shang J., Zhou X. (2020). Intraocular pressure changes and corneal biomechanics after hyperopic small-incision lenticule extraction. BMC Ophthalmol..

[27] Cao K., Liu L., Yu T., Chen F., Bai J., Liu T. (2020). Changes in corneal biomechanics during small-incision lenticule extraction (SMILE) and femtosecond-assisted laser in situ keratomileusis (FS-LASIK). Lasers Med. Sci..

[28] Qu Z., Li X., Yuan Y., Wang P., Li Y., Lin S., Lian H., Chen S., Ye Y., Wang J. (2024). In Vivo Corneal Biomechanical Response to Three Different Laser Corneal Refractive Surgeries. J. Refract. Surg..

[29] Abd El-Fattah E.A., El Dorghamy A.A., Ghoneim A.M., Saad H.A. (2021). Comparison of corneal biomechanical changes after LASIK and F-SMILE with CorVis ST. Eur. J. Ophthalmol..

[30] Yu M., Chen M., Dai J. (2019). Comparison of the posterior corneal elevation and biomechanics after SMILE and LASEK for myopia: A short- and long-term observation. Graefe’s Arch. Clin. Exp. Ophthalmol..

[31] He S., Luo Y., Ye Y., Chen P., Liu C., Lei L., Zhuang J., Yu K. (2022). A comparative and prospective study of corneal biomechanics after SMILE and FS-LASIK performed on the contralateral eyes of high myopia patients. Ann. Transl. Med..

[32] Xin Y., Lopes B.T., Wang J., Wu J., Zhu M., Jiang M., Miao Y., Lin H., Cao S., Zheng X. (2022). Biomechanical Effects of tPRK, FS-LASIK, and SMILE on the Cornea. Front. Bioeng. Biotechnol..

[33] Zarei-Ghanavati S., Jafarpour S., Hassanzadeh S., Bakhtiari E., Daraee G., Monadi S.D., Ziaei M. (2022). Changes in Corneal Biomechanical Properties After Small-Incision Lenticule Extraction and Photorefractive Keratectomy, Using a Noncontact Tonometer. Cornea.

[34] Lv X., Zhang F., Song Y., Zhai C., Guo N., Lai L., Xu Y. (2023). Corneal biomechanical characteristics following small incision lenticule extraction for myopia and astigmatism with 3 different cap thicknesses. BMC Ophthalmol..

[35] Liu M., Li N., Chen T., Tian G., Lin Y., Gao H., Shi W. (2023). Comparison of Corneal Biomechanics Treated with Femtosecond Laser-Assisted in Situ Keratomileusis and Small-Incision Lenticule Extraction by New Corneal Biomechanical Parameters of Corvis ST II. Cornea.

[36] Hashemi H., Roberts C.J., Elsheikh A., Mehravaran S., Panahi P., Asgari S. (2023). Corneal Biomechanics After SMILE, Femtosecond-Assisted LASIK, and Photorefractive Keratectomy: A Matched Comparison Study. Transl. Vis. Sci. Technol..

[37] Sedaghat M.R., Momeni-Moghaddam H., Yekta A.A., Maddah N., Roberts C.J., Savardashtaki M. (2024). Early elastic and viscoelastic corneal biomechanical changes after photorefractive keratectomy and small incision lenticule extraction. Int. Ophthalmol..

[38] Liu M., Shi W., Liu X., Li N., Chen T., Gao H. (2021). Postoperative corneal biomechanics and influencing factors during femtosecond-assisted laser in situ keratomileusis (FS-LASIK) and laser-assisted subepithelial keratomileusis (LASEK) for high myopia. Lasers Med. Sci..

[39] Guo H., Hosseini-Moghaddam S.M., Hodge W. (2019). Corneal biomechanical properties after SMILE versus FLEX, LASIK, LASEK, or PRK: A systematic review and meta-analysis. BMC Ophthalmol..

[40] Shen Y., Chen Z., Knorz M.C., Li M., Zhao J., Zhou X. (2014). Comparison of corneal deformation parameters after SMILE, LASEK, and femtosecond laser-assisted LASIK. J. Refract. Surg..

[41] Wu D., Wang Y., Zhang L., Wei S., Tang X. (2014). Corneal biomechanical effects: Small-incision lenticule extraction versus femtosecond laser-assisted laser in situ keratomileusis. J. Cataract. Refract. Surg..

[42] Elmohamady M.N., Abdelghaffar W., Daifalla A., Salem T. (2018). Evaluation of femtosecond laser in flap and cap creation in corneal refractive surgery for myopia: A 3-year follow-up. Clin. Ophthalmol..

[43] Yan H., Gong L.Y., Huang W., Peng Y.L. (2017). Clinical outcomes of small incision lenticule extraction versus femtosecond laser-assisted LASIK for myopia: A Meta-analysis. Int. J. Ophthalmol..

[44] Wang B., Zhang Z., Naidu R.K., Chu R., Dai J., Qu X., Yu Z., Zhou H. (2016). Comparison of the change in posterior corneal elevation and corneal biomechanical parameters after small incision lenticule extraction and femtosecond laser-assisted LASIK for high myopia correction. Cont. Lens Anterior Eye.

[45] Raevdal P., Grauslund J., Vestergaard A.H. (2019). Comparison of corneal biomechanical changes after refractive surgery by noncontact tonometry: Small-incision lenticule extraction versus flap-based refractive surgery—A systematic review. Acta Ophthalmol..

[46] Petsche S.J., Chernyak D., Martiz J., Levenston M.E., Pinsky P.M. (2012). Depth-dependent transverse shear properties of the human corneal stroma. Invest. Ophthalmol. Vis. Sci..

[47] Kamiya K., Shimizu K., Igarashi A., Kobashi H., Sato N., Ishii R. (2014). Intraindividual comparison of changes in corneal biomechanical parameters after femtosecond lenticule extraction and small-incision lenticule extraction. J. Cataract Refract. Surg..

[48] Agca A., Ozgurhan E.B., Demirok A., Bozkurt E., Celik U., Ozkaya A., Cankaya I., Yilmaz O.F. (2014). Comparison of corneal hysteresis and corneal resistance factor after small incision lenticule extraction and femtosecond laser-assisted LASIK: A prospective fellow eye study. Cont. Lens Anterior Eye.

[49] Damgaard I.B., Reffat M., Hjortdal J. (2018). Review of Corneal Biomechanical Properties Following LASIK and SMILE for Myopia and Myopic Astigmatism. Open Ophthalmol. J..

[50] Kanellopoulos A.J. (2018). Comparison of corneal biomechanics after myopic small-incision lenticule extraction compared to LASIK: An ex vivo study. Clin. Ophthalmol..

[51] Wei P., Cheng G.P., Zhang J., Ng A.L., Chan T.C., Jhanji V., Wang Y. (2020). Changes in Corneal Volume at Different Areas and Its Correlation with Corneal Biomechanics after SMILE and FS-LASIK Surgery. J. Ophthalmol..

[52] Jia Y., He R., Li X., Song Y., Wei J., Qin H., Yang X., Chen W. (2022). Effects of SMILE with different residual stromal thicknesses on corneal biomechanical properties of rabbits in vivo. J. Biomedical. Eng..

[53] Liu J., Wang Y., Zou H.H., Li M.D. (2021). Relation between corneal biomechanical alteration after small incision lenticule extraction and intraoperative cutting thickness. Chin. J. Ophthalmol..

[54] Damgaard I.B., Ivarsen A., Hjortdal J. (2018). Refractive Correction and Biomechanical Strength Following SMILE With a 110- or 160-μm Cap Thickness, Evaluated Ex Vivo by Inflation Test. Investig. Ophthalmol. Vis. Sci..

[55] Liu T., Yu T., Liu L., Chen K., Bai J. (2018). Corneal Cap Thickness and Its Effect on Visual Acuity and Corneal Biomechanics in Eyes Undergoing Small Incision Lenticule Extraction. J. Ophthalmol..

[56] El-Massry A.A., Goweida M.B., Shama A.E.S., Elkhawaga M.H., Abdalla M.F. (2015). Contralateral Eye Comparison Between Femtosecond Small Incision Intrastromal Lenticule Extraction at Depths of 100 and 160 μm. Cornea.

[57] Hosny M., Aboalazayem F., El Shiwy H., Salem M. (2017). Comparison of different intraocular pressure measurement techniques in normal eyes and post small incision lenticule extraction. Clin. Ophthalmol..

[58] Viswanathan D., Kumar N.L., Males J.J., Graham S.L. (2015). Relationship of Structural Characteristics to Biomechanical Profile in Normal, Keratoconic, and Crosslinked Eyes. Cornea.

[59] Liu Z.H., Li X., Zheng Z.Y., Lu Q. (2022). Correlation of the changes in corneal volume with the corneal biomechanical parameters after small incision lenticule extraction. Eye Sci..

[60] Ma J.N., Wang Y., Song Y., Shao T., Cai Y. (2019). The effect of corneal biomechanical properties on opaque bubble layer in small incision lenticule extraction (SMILE). Chin. J. Ophthalmol..

